# The effect of ICU-tailored drug-drug interaction alerts on medication prescribing and monitoring: protocol for a cluster randomized stepped-wedge trial

**DOI:** 10.1186/s12911-019-0888-7

**Published:** 2019-08-13

**Authors:** T. Bakker, J. E. Klopotowska, S. Eslami, D. W. de Lange, R. van Marum, H. van der Sijs, E. de Jonge, D. A. Dongelmans, N. F. de Keizer, A. Abu-Hanna

**Affiliations:** 1 Department of Medical Informatics, Amsterdam UMC (location AMC), Amsterdam, The Netherlands; 2 Pharmaceutical Research Center, School of Pharmacy, Mashhad University of Medical Sciences, Mashhad, Iran; 3 Department of Intensive Care and Dutch Poison Information Center, University Medical Center Utrecht, University Utrecht, Utrecht, The Netherlands; 4 Department of Geriatrics, Jeroen Bosch Hospital, s-Hertogenbosch, The Netherlands; 5 Department of General Practice and Elderly Care Medicine, Amsterdam UMC (location VUmc), Amsterdam, The Netherlands; 6 Department of Hospital Pharmacy, Erasmus MC, University Medical Center, Rotterdam, The Netherlands; 7 Department of Intensive Care, Leiden University Medical Center, Leiden, The Netherlands; 8 Department of Intensive Care Medicine, Amsterdam UMC (location AMC), Amsterdam, The Netherlands

**Keywords:** Intensive care, Drug-drug interactions, Computerized decision support systems, Alert fatigue, Stepped-wedge trial

## Abstract

**Background:**

Drug-drug interactions (DDIs) can cause patient harm. Between 46 and 90% of patients admitted to the Intensive Care Unit (ICU) are exposed to potential DDIs (pDDIs). This rate is twice as high as patients on general wards. Clinical decision support systems (CDSSs) have shown their potential to prevent pDDIs. However, the literature shows that there is considerable room for improvement of CDSSs, in particular by increasing the clinical relevance of the pDDI alerts they generate and thereby reducing alert fatigue. However, consensus on which pDDIs are clinically relevant in the ICU setting is lacking. The primary aim of this study is to evaluate the effect of alerts based on only clinically relevant interactions for the ICU setting on the prevention of pDDIs among Dutch ICUs.

**Methods:**

To define the clinically relevant pDDIs, we will follow a rigorous two-step Delphi procedure in which a national expert panel will assess which pDDIs are perceived clinically relevant for the Dutch ICU setting. The intervention is the CDSS that generates alerts based on the clinically relevant pDDIs. The intervention will be evaluated in a stepped-wedge trial. A total of 12 Dutch adult ICUs using the same patient data management system, in which the CDSS will operate, were invited to participate in the trial. Of the 12 ICUs, 9 agreed to participate and will be enrolled in the trial. Our primary outcome measure is the incidence of clinically relevant pDDIs per 1000 medication administrations.

**Discussion:**

This study will identify pDDIs relevant for the ICU setting. It will also enhance our understanding of the effectiveness of alerts confined to clinically relevant pDDIs. Both of these contributions can facilitate the successful implementation of CDSSs in the ICU and in other domains as well.

**Trial registration:**

Nederlands Trial register Identifier: NL6762. Registered November 26, 2018.

## Background

Drug-drug interactions (DDIs) are an important cause of adverse drug events (ADEs) [1]. In ICU patients, approximately 16% of all ADEs are caused by a DDI [2]. ADEs in the ICU are linked to increased length of stay, higher morbidity and mortality and increased hospital costs [3]. A DDI occurs when one or more drugs affect the pharmacokinetics (the body’s effect on the drug) and/or pharmacodynamics (the drug’s effect on the body) of one or more other drugs [4]. In the ICU, between 46 and 90% of the patients are exposed to a potential DDI (pDDI) [5]. This rate is twice as high compared to patients on general wards [6, 7]. A pDDI is defined as two potentially interacting medications administered concomitantly [8]. A pDDI may lead to an actual DDI, which could result in an ADE.

Studies have demonstrated the potential of clinical decision support systems (CDSSs) in preventing pDDIs [9, 10]. CDSSs focus on helping clinicians to improve their clinical performance. Computerized clinical decision support is defined as “providing clinicians or patients with computer-generated clinical knowledge and patient-related information, intelligently filtered or presented at appropriate times, to enhance patient care” [11]. A CDSS provides guidance at the point of prescribing by means of usually interruptive medication alerts that warn the prescriber for risky situations such as pDDIs [10]. However, the literature shows that there is considerable room for improvement, especially regarding the clinical relevance (and thereby the specificity) of the medication alerts generated by CDSSs [12–14]. Which pDDI alerts are generated by a CDSS depends on the underlying (sometimes commercial) knowledge base. [15] Lack of a fit between the clinical setting and the pDDI knowledge base used can be a cause of low specificity of medication alerts. Low specificity leads to alert fatigue and a high override rate of alerts. [16] Studies show that clinicians override alerts, including pDDI alerts, in 49 to 96% of the cases [16, 17]. These “side effects” of CDSS diminish the potential value of CDSSs for medication safety.

The ICU setting differs from other in- and outpatient settings for several reasons: patients in the ICU are more vulnerable for DDIs due to often-present impaired absorption, diminished renal and hepatic function, and polypharmacy [18]. On the other hand, as ICU patients are under continuous monitoring, some pDDIs are more acceptable than for non-ICU patients, because continuous monitoring allows for effective and timely risk management. Therefore, pDDI alerts that are clinically relevant in non-ICU settings may be of limited clinical value in the ICU. However, consensus on which pDDIs are clinically relevant in the ICU setting is lacking, and this hampers adequate pDDI alerting through CDSSs.

Therefore, the primary aim of this study is to evaluate the effect of tailoring pDDI alerts to the ICU setting on pDDI prevention among Dutch ICUs. To this end, we first identify which pDDIs are considered clinically relevant for the ICU setting through a standardized and rigorous Delphi procedure with a national expert panel. Next, we will use the resulting set of clinically relevant pDDIs as the ICU-specific pDDI knowledge base that a CDSS will use to generate alerts. Subsequently we will evaluate the effectiveness of this tailored strategy on the prevention of the clinically relevant pDDIs.

### Theoretical foundation

**Fig. 1:**
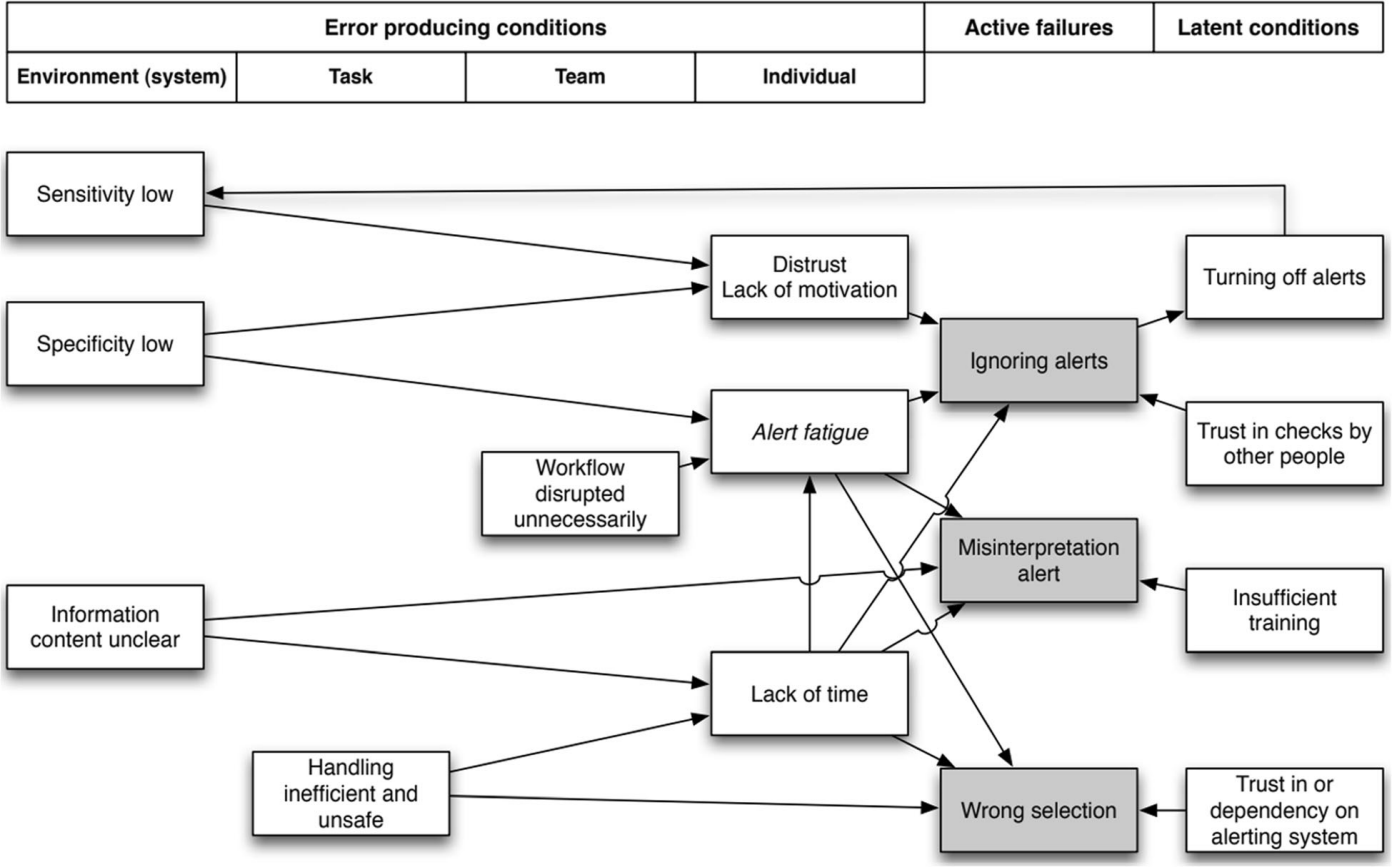
Adapted version Reason’s model of accident causation applied to drug safety alerting from van der Sijs et al. (with permission) [16, 20]

Alert fatigue can be described as “the mental state that is the result of alerts consuming too much time and mental energy, which can cause relevant alerts to be unjustifiably overridden along with clinically irrelevant ones” [16, 19]. Alert fatigue may lead to patient harm, when alerts pertinent to patient safety are overlooked and ignored [10]. To combat alert fatigue, it is important to optimize the balance between sensitivity and specificity of an alerting system [10, 12, 16]. The sensitivity of an alerting system is the ability of the system to produce an alert when that is necessary. When sensitivity is high, there are only few situations in which the system fails to provide an alert when it should have provided one. The specificity of an alerting system is the ability of the system to produce an alert, only if necessary. When the specificity is high, the system produces few unnecessary alerts (‘false alarm’) [13, 16]. To visualize sensitivity and specificity influencing alert fatigue and alert overrides, van der Sijs et al. [16, 20] applied Reason’s model of accident causation to drug-safety alerting and used this model to interpret how errors arise in an alerting system. The model is shown in Fig. 1.

Lack of a fit between the clinical setting (e.g. ICU setting) and medication alerts is one cause of low specificity. Therefore, we hypothesize that using a pDDI knowledge base not tailored to the ICU setting, would lead to alert fatigue and thereby to ignoring pDDI alerts, as is depicted in Fig. 1. This in turn may result in also overriding clinically relevant pDDI alerts, compromising medication safety in the ICU. Therefore, to improve the specificity of pDDI alerts for the ICU setting, we first need to assess which pDDIs are clinically relevant for the ICU setting. A tailored pDDI knowledge base for the ICU setting will increase the specificity of pDDI alerts and reduce alert fatigue and pDDI alert overrides. This in turn will decrease the risk of ignoring relevant alerts, and eventually decrease the number of pDDIs.

## Methods/design

### Study design

To evaluate the effect of tailoring the pDDI knowledge base to the ICU setting on pDDI prevention we will implement a trial with a stepped-wedge design. A stepped-wedge trial is a type of cluster trial in which an intervention is rolled-out sequentially to the clusters over a number of periods that are called ‘steps’ [21]. By the end of the trial period, all clusters will have received the intervention. The order in which the clusters receive the intervention is randomized [22]. Data analysis to determine the overall effectiveness of the intervention subsequently involves comparison of the data points in the control section of the wedge with those in the intervention section [21].

We prefer a stepped-wedge design over a randomized controlled trial because withholding CDSS from those already using it is not considered ethical in our study. Withholding decision support in an environment where physicians are used to receive support to prevent pDDIs could have a negative effect on patient safety. Furthermore, methodologically speaking the stepped-wedge design is comparable to a parallel cluster RCT. Also, when the clusters are large, as is the case in our study, a stepped-wedge design is more powerful than a parallel design [23].

The order in which the ICUs will receive the intervention will be randomized, because randomization helps reduce the risk of bias and thereby increase the internal validity of the study [24]. An independent researcher who is not involved in further conduct of this study will perform the computerized randomization. As all the prescribing physicians in the participating ICUs and the investigators will be aware of the ‘step’ from control to intervention status, blinding is not feasible. Further details on the implementation of the stepped-wedge design in this study can be found in the paragraph ‘Stepped-wedge design implementation’.

This protocol is reported in accordance with the SPIRIT 2013 guideline for content of clinical trial protocols [25]. The results of this study will be reported in accordance with the CONSORT statement [26].

### Study setting

The setting of this study is the Dutch intensive care. In the Netherlands, all ICUs are mixed medical-surgical closed-format ICUs. All Dutch ICUs participate in the Dutch quality registry called National Intensive Care Evaluation (NICE). The NICE registry offers ICUs feedback and benchmarking on patient outcomes, including mortality, and allows them to compare their outcomes with those achieved nationally and by groups of similar hospitals [27].

To participate in our study, we require that an ICU department should use the commercial patient data management system, MetaVision ICU (*i*MD*Soft*®) during the whole trial. This is a pragmatic criterion, but one that allows a straightforward integration of the intended CDSS within this system. In particular, our CDSS will be obtained by adapting a current version of a CDSS that already works with MetaVision ICU but which provides pDDI alerts indiscriminately, hence regardless of clinical relevance for the ICU setting. In effect we need to replace the existing pDDI knowledge base in this CDSS to one which is tailored to the ICU setting. This essentially means that we will make a critical selection of the pDDIs for which the alerts should fire. By opting for one type of data management system and the CDSS that is able to operate within it, all participating ICUs will receive the pDDI alerts in the same manner. We invited all ICUs that use MetaVision, 12 in number, to participate in this study via the network of the NICE registry. Nine ICUs have agreed to participate in the trial and have provided an intensivist of the ICU as a local contact person. The remaining three ICUs decided not to participate in the trial because their hospital was in the process of migration to a different, hospital-wide, patient data management system. The nine participating ICUs have an overall capacity of 156 beds, and in total around 11200 (median: 854; IQR: 793–1785) patients are admitted yearly. One ICU is academic, eight are non-academic. The ICUs are geographically well distributed over the Netherlands.

The CDSS system that is compatible with MetaVision ICU, and available since January 2012, is the Medication Interaction Module (MiM, It*é*Medical®). The use of MIM within MetaVision ICU is optional. Five out of the nine participating ICUs already use MiM in Metavision ICU for four to six years before the start of the trial. The MiM can produce two types of medication alerts at the point of prescribing: pDDI alerts and duplicate orders alerts. Each ICU can set the (severity) level at which MiM provides alerts. The pDDI knowledge base integrated in MiM is the so-called G-standard. The G-standard is an evidence-based professional database for the management of medication alerts, developed and maintained by a working group of the Scientific Institute of Dutch Pharmacists. [8] The G-standard is used in most Dutch hospitals as the underlying knowledge base for decision support. For each pDDI, the working group assigns a severity and evidence level based on Summary of Product Characteristics and literature. Furthermore, the working group also provides a summary of interaction mechanism and recommendations on how to handle medication alerts (e.g. by monitoring of laboratory values). This situation is unique, as in many other countries, the development and maintenance of CDSS knowledge bases for medication are not organized at a national level. In the G-standard, medications are represented by generic product codes. The generic product code describes medication on the pharmaceutical level, based on the following pharmaceutical characteristics: active ingredients, strength, dosage form and route of administration [28]. All pDDIs are enlisted in the G-standard as pairs of generic product codes. The G-standard however originates from the outpatient setting and is not tailored to the ICU setting.

### Patient eligibility criteria

Patients over the age of 18 years admitted to the ICU having any administered medications during their ICU admission will be included.

### Establishing clinically relevant pDDIs

To determine which pDDIs are clinically relevant for the ICU setting, we will apply a modified Delphi procedure, in which an expert panel of intensivists and hospital pharmacists will assess the clinical relevance of pDDIs. The pDDIs to be assessed will be selected from the G-standard according to their frequency in the ICU and the assigned severity level. In essence, a pDDI is defined as clinically relevant for the ICU setting when non-standard monitoring for possible pDDI effects is required, or the pDDI should always be avoided. The modified Delphi procedure will be based on the RAND method [29], and will consist of an online questionnaire and expert panel meeting.

### Intervention

Our intervention consists of a version of the MiM that will only provide alerts for pDDIs considered clinically relevant in the ICU, according to our Delphi procedure. pDDI alerts considered not clinically relevant for the ICU will be turned off. This MiM version tailored to the ICU setting will be called MiM+. Our hypothesis is that alert fatigue can be reduced by MIM+, as the set of pDDIs for which alerts are provided at the point of prescribing, will be restricted and more specific for the ICU setting.

In the four participating ICUs that do not use the current MIM CDSS for pDDIs, the MiM+ software will be installed. Duplicate order alerts will not be shown in these four ICUs.

In the five ICUs that already use MiM as CDSS for pDDIs, MiM+ will start operating in these ICUs during the intervention phase. However, pDDI alerts not assessed during our Delphi procedure and for which an ICU gets alerts according to its current alert level setting, will not be changed. Furthermore, other types of alerts such as duplicate order alerts will also not be changed by our intervention. In short, the ICUs with a MiM CDSS will experience change in the alerts that were assessed in the Delphi rounds, in the sense that alerts for the pDDIs deemed irrelevant will be suppressed. All participating ICUs will receive an on-site training for the prescribing physicians about MiM+ given by a researcher involved in this study.

Apart from this intervention, all participating ICUs will receive two performance feedback rapports before MiM+ implementation. In these reports, results of a retrospective analysis of medication administration data on the occurrence of pDDIs will be included. The results will include pDDI occurrence over one year for the specific ICU, benchmarked against the other participating ICUs.

### Qualitative evaluation

**Fig. 2:**
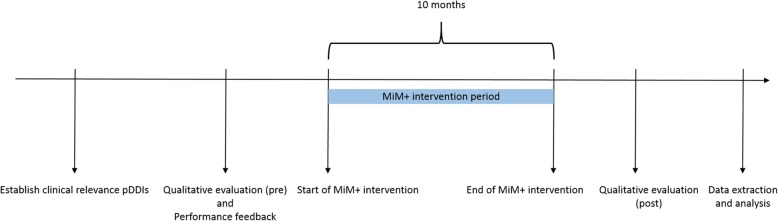
Timeline of different components of this study

Success of CDSS interventions is partly dependent on human factors such as user expectations and acceptability, and system usability [30]. Therefore, in order to better understand why MIM+ worked or not afterwards, we also will conduct a qualitative evaluation before and after MiM+ implementation. For this evaluation, a mixed method approach will be used including semi-structured interviews with users, observations of users handling pDDI alerts and a survey among ICU physicians. In Fig. 2, an overview of this study over time can be found.

### Stepped-wedge design implementation

**Fig. 3:**
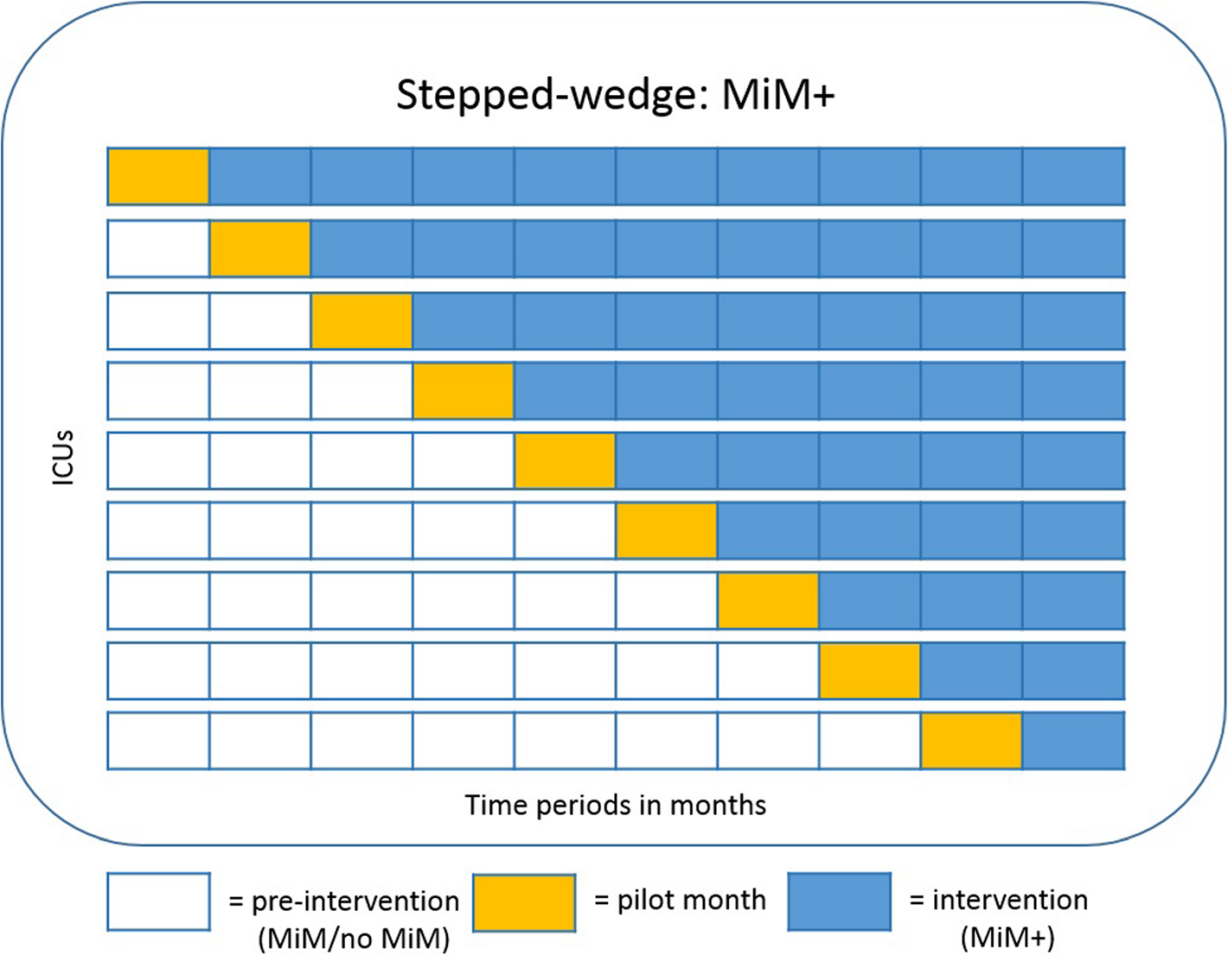
Timeline of stepped-wedge trial

In our study, each ICU represents one cluster and the MIM+ is rolled-out over nine steps of one month each (see Fig. 3). At each step, one cluster will start with the MIM+. Maximum power for a given number of clusters is achieved when each cluster has its own step [22]. The total duration of the trial is ten months. Whenever an ICU starts with the MIM+, the first month will be considered a pilot month, in which small, local changes to the MiM+ will be allowed. For example, an ICU could argue for the inclusion of an alert for an extra pDDI that was not included in the MiM+ or to discard a pDDI alert from the MiM+. This will be allowed, because the type of patients and medications can differ between ICUs and this could lead to different needs for pDDI alerts. We hypothesize that such local limited tweaking will rarely happen but can positively influence system acceptance [30].

### Study outcomes

The primary outcome of this study is the incidence of clinically relevant pDDIs per 1000 medication administrations. To be able to compare our results to other studies, we also defined secondary outcomes. These are the number of pDDIs per patient, the number of clinically relevant pDDIs per patient, the proportion of patients admitted to the ICU with at least one pDDI, and the proportion with at least one clinically relevant pDDI [9]. Other secondary outcomes are ICU length of stay, the override rate of clinically relevant pDDI alerts and the number of ADEs related to DDIs per 1000 medication administrations. In Table 1 definitions of study outcomes are described. These definitions are in line with previous studies [5, 8].

**Table 1:** Overview of study outcomes

Study outcomes	Definition
pDDI	Two potentially interacting medications administered concomitantly which could result in an actual DDI.
Clinically relevant pDDI	A pDDI considered clinically relevant for the ICU setting, according to the results from our Delphi procedure.
ADE related to DDI	A DDI that resulted in patient harm.
Appropriately handled pDDI	Two potentially interacting medications were administered concomitantly (pDDI), but there is evidence that adequate measures were taken to prevent patient harm or diminish the risk of patient harm.

In addition, we will also measure the proportion of appropriately handled clinically relevant pDDIs. This is an important outcome, specifically in the ICU setting, where the patient is in a critical condition and sometimes the risk of prescribing interacting medications is subject to the need for treatment [17]. Furthermore, if prescribing a pDDI cannot be averted, there are several management strategies to keep the potential harm to a minimum. Management strategies include for example monitoring laboratory values such as creatinine, potassium, therapeutic drug monitoring and adjustment of medication dosage [8]. Because of this, it is not only important to know whether a reduction in the number of clinically relevant pDDIs has occurred, it is also important to know how many pDDIs occurred that were handled appropriately, for example by conducting therapeutic drug monitoring.

To assess whether a clinically relevant pDDI alert is handled appropriately or not, for each clinically relevant pDDI we will list the possible management strategies based on the recommendations available in the G-standard. A pDDI will be considered as handled appropriately if there is ‘evidence in the data’ that one or more of the defined management strategies has been implemented based on medication, laboratory and ECG data of the patient.

### Data collection

To assess the effect of our intervention on the primary and secondary outcomes, and to gain insight in pDDI type and occurrence for the purposes of our Delphi procedure, we will use routinely recorded data extracted from MetaVision ICU, MiM/MiM+, and the NICE registry.

Data covering all ICU admissions in the period from at least one month prior to the MIM+ implementation up to one month after the intervention period will be extracted. All data from Metavision ICU and MiM/MiM+ will be collected at the patient level using a coded admission number of the patient as an identifier. This coded admission number cannot be used to identify a specific patient. The following routinely recorded data from Metavision ICU will be extracted: medication orders, orders for an electrocardiogram (ECG) and laboratory orders. Medication orders will include the name of the ordered medication, generic product code of the medication, dosage and frequency, validation of the order indicating whether the medication order was actually administered, the start and end time of the order, and whether or not the order was cancelled. In MetaVision ICU, each administration is registered separately. These data are necessary to assess the primary outcome: the occurrence of clinically relevant pDDIs per 1000 medication administrations. Laboratory orders will include name of test ordered, date and time of the order, and for results of the test. ECG orders will include date and time of the order. ECG and laboratory data together with medication data are necessary to assess if, following a pDDI alert, appropriate management strategies were employed (secondary outcome: appropriately handled clinically relevant pDDIs) and to assess whether ADEs occurred (secondary outcome: number of ADEs related to DDIs per 1000 medication administrations).

The following routinely recorded data from MiM and subsequently MiM+ will be extracted: all (clinically relevant) pDDI alerts that were produced, date and time of the alert, which medication (name and generic product code) was involved, if the alert was overridden or not, and if entered by the user, the reason for overriding the alert. These data are necessary to assess the secondary outcome: override rate of (clinically relevant) pDDI alerts per 1000 medication administrations.

To detect (clinically relevant) pDDIs in the medication orders data, we will develop a computerized algorithm based on the pDDI rules from the G-standard. This algorithm will then be applied to medication order data from the participating ICUs. Only validated medication orders, i.e. medication orders that were actually administered, will be considered. In addition, each separate administration for a specific drug will be attributed to one composite medication administration if the time gap between separate administrations does not exceed a specific amount of time, such as 24 h (regardless of the route of administration).

A second developer will validate the algorithm. To assess the number of ADEs related to DDIs per 1000 medication administrations, we will develop computerized ADE triggers. Triggers are sentinel words or events that may point to the occurrence of an ADE, such as increased or decreased laboratory values, ordering an ECG or prescribing an antidote [31]. The triggers that we develop and ADEs identified by these triggers will be published along with the results of this study.

In addition to data from Metavision ICU and MiM/MiM+, we will also use data submitted to the NICE registry by the participating ICUs. These data include clinical, physiological and demographic data required to calculate outcomes such as ICU length of stay, hospital length of stay, hospital mortality, and expected mortality based on disease severity predicted from clinical data of the first 24 h of ICU admission [27]. Data from Metavision ICU, MiM/MiM+ can be linked with NICE registry data using the earlier mentioned unique coded admission number.

### Data validation

All extracted data will be validated. Validation consists of automated checking for missing values and outliers. Missing values will be reported in the results. Outliers will be investigated and in case of obviously incorrect measurement or entry mistake, outliers will be dropped. Only data that can be linked to the NICE database will be included in the analysis, but in essence every admission in any of the participating ICUs should correspond to an admission in the NICE registry.

### Sample size

To calculate power of our stepped-wedge trial, the statistical software PASS 15.0.4 (NCSS LLC., Kaysville, UT) was used. The calculation considers the following factors: the number of steps, the size of the steps, the number of clusters per step, the number of medication administrations per cluster per step, the anticipated improvement of the intervention, the estimated event rate, and the estimated intra-cluster correlation (ICC).

The anticipated improvement was defined as the overall difference in the number of clinically relevant pDDIs per 1000 medication administrations before and after the MiM+ implementation. We estimate an average relative reduction of 20% in all participating ICUs. According to a systematic review assessing effects of IT-based interventions on outcomes related to DDIs, relative reductions ranging from 15 to 29% have been reported [9].

Based on preliminary analysis of medication data extracted from 5 out of the 9 participating ICUs and one ICU that does not participate in the MiM+ component of the intervention, the number of clinically relevant pDDIs per 1000 medication administrations was estimated to be 42.0 (event rate). With a relative reduction of 20%, the event rate after the intervention is therefore estimated to be 33.6. As an indicator of face validity, a senior intensivist who is involved in the study, considered that a 20% relative reduction from 42.0 is clinically relevant. With a sample of 9 clusters in a stepped-wedge cluster-randomized design with 10 time periods, 9 steps, and one cluster switching from control to intervention at each step and an average number of 6000 medication administrations per cluster, with an average of 600 medication administrations per cluster per time period, calculations show 83% power to detect a relative reduction of 20%, considering an estimated intra-cluster correlation of 0.12. The calculations are based on the Poisson distribution, the test statistic used is the two-sided Wald Z-Test and the significance level of the test is 0.05.

### Statistical analysis

Depending on the distribution of variables, descriptive statistics will be presented as mean and standard deviation, median and IQRs, or percentage as appropriate. Continuous variables will be compared using the t-test or Mann-Whitney test. Categorical variables will be compared with a Chi squared test or Fisher’s exact test. To assess the effect of tailoring pDDI alerts to the ICU setting on the number of pDDIs per 1000 medication administrations we will analyse data from the stepped-wedge trial, adjusting for data clustering and for temporal trends if necessary. A generalized linear model will be fitted with correction for clustering via generalized estimating equations. To adjust for possible confounding patient characteristics (age and disease severity) and other variables (such as the previous use of MiM prior to MiM+) will be considered for addition in the model as covariates. We anticipate that most DDIs will occur around the third day of admission, because by that time chronic home medication often is restarted in the ICU while medication typically prescribed in the ICU is still prescribed [32].

All analyses will be performed according to the intention-to-treat principle. For hypothesis testing a probability level of less than 0.05 will be considered statistically significant. All statistical tests will be two-sided. The R statistical software environment will be used for the analyses.

### Harm

We do not anticipate any harm to the study population caused by our interventions.

### Protocol amendments

Important protocol modifications will be communicated to the appropriate parties, such as the participating ICUs, trial registry or the Medical Ethics Committee.

### Confidentiality

Only pseudonymized data will be extracted from MetaVision ICU, the MiM and MiM+. For that purpose, each ICU admission will be assigned a coded admission number as a unique identifier. This number is meaningless outside the ICU and the NICE registry. With each participating ICU we will draw an agreement considering data sharing and data extraction arrangements. All data will be stored according to data management policy prevailing in our organization and will be accessible only by authorized trial investigators.

### Dissemination policy

Study results will be communicated via scientific publication and presentations during conferences as well as via newsletters to the participating ICUs.

## Discussion

Our study has several strengths. First, this is a large multi-center study with a stepped-wedged randomized controlled study design. In this study, different types of data are combined, such as medication data and laboratory data and these data can be linked with data from the NICE registry. In addition to our primary outcome, we use secondary outcomes and are able to compare our results with other studies. Moreover, besides counting the number of pDDIs, we investigate whether or not the pDDI was handled appropriately. The stepped-wedge design is a powerful design that is comparable to a parallel cluster randomized controlled trial design. When the number of measurements within the clusters are large, as in our case, the stepped-wedge design will be more powerful than a parallel design [23]. Second, by involving future users of the MiM+ in the Delphi procedure to assess which pDDIs are considered clinically relevant in the ICU setting, acceptance of the MiM+ is more likely [30, 33]. Third, by using routinely registered data, this study imposes no extra registration burden to the ICUs and the data reflects daily practice. Fourth, the effectiveness of sociotechnical implementations depends on the organizational and cultural setting in which it is implemented. [30] By performing a qualitative evaluation, we will have a better ability to explain the effects of tailoring pDDI alerts to the ICU setting. Lastly, our hypothesis that tailoring pDDI alerts to the ICU setting will improve pDDI prevention is founded on Reason’s model of accident causation applied to drug-safety alerting [16]. By using a theoretical model, we build on existing knowledge and are able to explore the underlying mechanisms of alert effectiveness described in this theory.

There are also limitations. Adjusting the pDDI knowledge base of a CDSS is not the only factor that has potential to reduce alert fatigue. There are other factors influencing alert fatigue, such as timing and design of the alert, which will not be investigated in this study [13]. However, as all ICUs in this study will use the same CDSS, we expect these other factors to be equal in all participating ICUs. Furthermore, the presence of other alerts, such as duplicate order alerts, may increase alert fatigue and thereby potentially diminish the effect of our intervention.

Lastly, consensus methods such as our Delphi procedure contain certain methodological issues such as bias in the selection of participants, and subjectivity in the judgements of expert panel members. To ensure experts involved are representative for the Dutch ICU care, we will not only invite experienced intensivist and hospital pharmacists from the participating hospitals but also from Dutch hospitals not participating in the trial. By using a scoring instrument and live panel discussion, we aim to limit subjectivity in judgments about clinical relevance of pDDIs.

As with cluster randomized trials in general, stepped-wedge designs require larger sample sizes because patients may be similar within one cluster. Even though randomization improves the balance of important characteristics across study arms, with a small number of clusters as in our study, such a balance cannot be ensured [22]. A second methodological limitation is the potential for confounding by temporal trends. Comparisons of outcomes between earlier and later periods may be influenced by background changes that affect the outcome of interest, irrespective of the intervention [22]. We believe that given the relatively short study period of 10 months this risk is limited.

Although in this study, for practical reasons, we use specific CDSS software (MiM+), the knowledge gained about which pDDIs are clinically relevant to the ICU can be used in any other type of CDSS. Therefore, if successful, implementing our set of clinically relevant pDDIs should be beneficial in other ICUs, also outside the Netherlands, as frequently occurring pDDIs in the ICU setting seem comparable between countries [34]. Our results may also encourage caregivers from other settings, and developers of CDSSs, to establish a pDDI knowledge base for a specific setting of patient group, for example for pediatric or oncology care.

## Conclusion

Use of CDSSs to aid clinicians in safe medication prescribing is becoming standard in healthcare [9]. However, CDSSs still have important limitations, notably to alert fatigue and high override rates [3, 10]. The sensitivity and specificity associated with the underlying knowledge base used in the CDSS are important factors linked to alert fatigue and hence to CDSS effectiveness [12]. Our trial will contribute to the current knowledge and understanding of the effectiveness of CDSS on, specifically, pDDI prevention in the ICU setting, and more globally to the potential gains obtained by improving the specificity of alerts in other settings. Our results may contribute to knowledge necessary for successfully optimizing CDSSs for pDDI prevention and other domains.

## Data Availability

Not applicable / Data sharing is not applicable to this article as no datasets were generated or analysed during the current study.

## Abbreviations

ADE: Adverse drug event
CDSS: Clinical decision support system
DDI: Drug-drug interactions
ECG: Electrocardiogram
ICC: Intra-cluster correlation
ICU: Intensive care unit
MiM: Medication Interaction Module
MiM+: Medication Interaction Module tailored to the ICU setting
NICE: National Intensive Care Evaluation
pDDI: Potential drug-drug interaction
